# Remarks on Atmospheric Plates

**Published:** 1852-07

**Authors:** T. L. Buckingham

**Affiliations:** Dentist.


### ARTICLE XIV.

*Remarks on Atmospheric Plates.* By T. L. Buckingham, M. D., Dentist.

Recently, there has been much discussion respecting the use of the cavity or chamber in plates. Various opinions have been expressed, some approving, others disapproving, of its use. Some consider it the best method of inserting full upper sets of artificial teeth, and have adopted it not only in these cases, but in some partial sets also, while others are as strongly opposed to it. They think that, instead of its being an advantage, it is a disadvantage ; that, although it will assist to hold up a plate at first, the cavity soon becomes filled by the congested vessels, and then, instead of assisting to keep the plate in its place, it only tends to displace it.

We propose, in this article, to attempt to explain the manner in which it acts, and answer some of the objections made to its use. We own we are its advocate : we have used it in numerous instances, and do not know how we could get along without it, unless something can be discovered that will answer better. We are certainly not disposed, at present, to go back to the old mode of making plates, no wider than will cover the alveolar ridge, and compel the wearers to keep their teeth closed while they talk, and hold them up with their tongue when they want to take anything into the mouth.

We will first state that the same depth of cavity will not suit for all cases. For a soft, spongy mouth, the cavity should be deeper than for one that is hard and unyielding. In the first, the mucous membrane will bear stretching to a considerable extent, without producing irritation ; in the other, if the cavity is deep, there will be a constant vacuum, which will produce so much strain upon it, as to cause a very unpleasant, if not ultimately an injurious, effect. It is not at all uncommon to find mouths in which the mucous membrane has been disrupted, by the constant pressure made upon it, and an abnormal growth of tissue filling the cavity entirely up, which prevents it from being of any further use.

We never make a cavity deeper than the mucous membrane will stretch and fill up, without irritation, depending upon the elasticity of the vessels to hold it in its place ; for, it takes as much pressure to keep the vessels congested, as it did to congest them at first, unless they have become permanently enlarged, which seldom is the case, if there has not been too much strain upon them.

The question is asked in the January number of the "American Journal of Dental Science," how we "explain, upon scientific principles, how a partial vacuum can be obtained?" That a partial vacuum can be produced in the mouth, we suppose nobody will doubt. Every time we inspire, or every time we drink, (unless it is when the head is thrown back, and the fluid poured into the mouth,) proves that there is a vacuum, and the air or fluid rushes in to fill it. But this is not vacuum enough to be of any benefit to us ; we can produce a greater vacuum by what we term "suction."

If we take a tube, closed at one end, and put the open end in the mouth, close the lips and nostrils, then, by the action of the thoracic muscles, produce a partial vacuum in the chest, a portion of the air in the mouth rushes into the lungs, to establish an equilibrium. This is what is termed suction. Now, if the tongue be pressed on the open end of the tube, so as to close it, and the air admitted into the mouth, the tube will stick to the tongue, and it will require a considerable effort to pull it off. This, every child of five years of age has done a hundred times with its mother's keys or thimble ; and this is vacuum enough for our purpose. It is not equal to fifteen pounds to the square inch, we admit ; nor, perhaps, one-fifth of it. Fifteen pounds to the square inch would tear the mucous membrane from the palatine bones; it would cause the blood to ooze out through the pores of the skin. The thickest part of a bullock's hide would hardly bear it, over any extent of surface; and yet we talk about applying that amount of pressure to the delicate membrane in a lady's mouth! Fifteen pounds is nearly a perfect vacuum at the level of the sea. This is more than we can obtain with an air-pump; for, there must always be air enough left in the receiver to work the valves, even if we allow nothing for leakage.

That we can produce a partial vacuum in the mouth being proved, the question is asked, "how does the air get out of the chamber without having a valve in it?" It would have been much more difficult to answer, had it been asked how it could be "kept in." If we take an India rubber bag, and tie the mouth so as to prevent the escape of the air it contains, then place it under the receiver of an air-pump, and exhaust the air from the receiver, the bag will swell up to several time its ordinary size. If, in the place of an India rubber bag, we take a glass bottle, and cork it up tight, and place it under the receiver, the bottle, unless it is very strong, will be broken to pieces: for, the pressure of the air on the inside, is equal to the pressure taken off the out side. But how does the air get out from under the plate? We make a partial vacuum in the mouth; in doing which, the teeth are held some distance apart; the cheeks are pressed down upon the artificial teeth, which loosens them from the gum; then the air rushes out from under the plate, to establish an equilibrium with the air that is in the mouth and lungs. Just before the air is admitted into the mouth, the plate is pressed firmly against the roof, with the tongue, and held there. If the plate fits perfectly around the edges, the air is prevented from getting into the chamber; and by the pressure of the atmosphere on the outside, it is made to adhere firmly to its place.

Now that we have explained, or at least attempted to do so, how we get a vacuum in the chamber, we will say a few words on the chambers generally in use.

There are but three or four kinds of chambers which we think necessary to examine at present; all the others have either been abandoned, or are used so seldom, that we will not mention them here. The first we propose to notice, is made by enlarging the rugæ of the mouth, or sometimes by making a number of small chambers in the plate, about the size of half a pea, without regard to locality. With this kind of chamber, we have not succeeded very well, although others continue to use them, and in some cases, prefer them to all others. In our cases, we have found that, unless the edges of the plate fit perfectly, the air would creep in at some place, and destroy the adhesion. It is almost impossible to make those portions of the plate, between the chambers, fit so perfectly as to isolate each chamber. If this is not done, and we have to depend upon the edges of the plate to exclude the air, we can see no advantage possessed by this kind over one large chamber.

The next is Dr. Flagg's. This, we have also used, but without much success, finding the difficulty attending it which might have been expected from its position. The cavities are formed on the top of the alveolar ridge—the place, above all others, on which the plate should press in masticating. If we make a chamber here on each side, "about one inch in length, by three-eighths in width, and one-tenth in depth," as the doctor proposes, the plate forms a wedged-shaped cup, down the sides of which the gums are forced every time pressure is made upon it. The whole pressure comes upon the alveolar and maxillary bones on the outside, and the maxillary and palatine bones on the inside, and not upon the alveolar ridge; and we all know that an inclined plane will produce a greater pressure than the same amount of force applied in a direct line on a plain surface.

Dr. Cleavland's is the next to be noticed. This is made by striking up a plate to fit a cast of the mouth; an opening is then cut in the centre, varying from the size of a dime to that of a quarter of a dollar. Over this plate, and about an eighth of an inch from it, another plate is fitted, which meets the first as it rises on the alveolar ridge. Here the plates are soldered together; this makes a very smooth and pleasant surface for the tongue. These chambers are much larger on the inside than they are at the opening; this allows the gum, when it is forced up into the chamber, to curl over the edge of the plate. There is no doubt about this chamber holding a plate firm; but the edge of the plate irritates the gum so much as to keep it constantly sore. In addition to this, the shape of the chamber precludes the possibility of keeping it clean, and it becomes very offensive. To remedy this objection, a band is sometimes inserted around the external opening, and soldered to the plates. This makes a chamber exactly like Gilbert's with an acute edge; this sharp edge always keeps the gum sore. We have seen several cases where this chamber has been worn for some length of time, and they all presented the same appearance, namely: a ring of inflammation upon the gum, which, in some cases, extended over the whole roof of the mouth.

In inserting artificial teeth, we should produce as little irritation as possible. It is bad enough to be compelled to wear them, without having the mouth constantly sore. This chamber will hold a plate firmer than any other we know of. If the plate fits close around the edge of the chamber, it matters very little, whether it fits in other places or not. It is like a dry cup on some soft part: pull it in any direction you please, it still adheres.

The last chamber we will notice, is Gilbert's. This kind has been used in more cases than any other we know of, and has generally given more satisfaction. In his application for a patent, Gilbert specifies no size or shape, but claims as follows: "The plate being made single, and a chamber being sunk in the central part of the upper surface of the plate, in which a vacuum can be formed by the tongue." Whether Mr. Gilbert was the first to use this kind of a chamber, we do not know; but this we do know: that as soon as he made known to the profession that he had taken out a patent, many others, in all parts of the country, claimed to have used the same thing years before. This may appear strange to those out of the profession; but, when they consider that every invention that has been made in dentistry, has had at least a dozen claimants; they must conclude that every dentist is a genius, and the only reason his inventions have not been brought before the profession, is because he has not quite completed them! for, make what you will, and show it to all you meet, you will find they have all used, or thought of it, years before.

As we have stated before, this chamber should not always be made of the same depth. We sometimes make it with abrupt edges, by driving the edge down with a punch, (but never make it sharp enough to cut;) in other cases, the chamber gradually diminishes in depth from the centre to the edge. The only rules we have to govern us, is: first, not to make the edges of the chamber sharp enough to irritate the gum; and next, to make the cavity no deeper than the mucous membrane will fill up without producing inflammation.

When we insert artificial teeth, we should not try how great a weight we can make them sustain without falling; but to give the most satisfaction and comfort to our patients. To do this, it is not necessary that it should take fifteen or twenty pounds to displace them. All that is required, is, that they shall adhere tight enough to prevent them from falling when the mouth is opened. If they do this, and the teeth are articulated properly with the under teeth, there is very little danger of their coming down in mastication, after the patient has worn them a short time.—*Dental News Letter.*

